# Emergence of Carbapenem-Resistant *bla*_POM-1_ Harboring *Pseudomonas otitidis* Isolated from River Water in Ghana

**DOI:** 10.3390/antibiotics14010050

**Published:** 2025-01-08

**Authors:** Frederick Ofosu Appiah, Samiratu Mahazu, Isaac Prah, Taira Kawamura, Yusuke Ota, Yohei Nishikawa, Mitsunori Yoshida, Masato Suzuki, Yoshihiko Hoshino, Toshihiko Suzuki, Tomoko Ishino, Anthony Ablordey, Ryoichi Saito

**Affiliations:** 1 Department of Molecular Microbiology and Immunology, Institute of Science Tokyo, Tokyo 113-8510, Japan; fredo.vip@tmd.ac.jp (F.O.A.); smahazu@noguchi.ug.edu.gh (S.M.); prahmolv@tmd.ac.jp (I.P.); ma220029@tmd.ac.jp (T.K.); y-ota.micr@tmd.ac.jp (Y.O.); 2 Department of Parasitology and Tropical Medicine, Institute of Science Tokyo, Tokyo 113-8510, Japan; tishino.vip@tmd.ac.jp; 3 Computational Bio Big-Data Open Innovation Laboratory, AIST-Waseda University, Tokyo 169-0082, Japan; yohei-nishikawa@aist.go.jp; 4 Research Organization for Nano & Life Innovation, Waseda University, Tokyo 162-8644, Japan; 5 Department of Mycobacteriology, Leprosy Research Center, National Institute of Infectious Diseases, Tokyo 162-8640, Japan; m-yoshida@niid.go.jp (M.Y.); yhoshino@niid.go.jp (Y.H.); 6 Antimicrobial Resistance Research Center, National Institute of Infectious Diseases, Tokyo 162-8640, Japan; suzuki-m@niid.go.jp; 7 Department of Bacterial Pathogenesis, Tokyo Medical and Dental University, Tokyo 113-8510, Japan; suzuki.bact@tmd.ac.jp; 8 Department of Bacteriology, Noguchi Memorial Institute for Medical Research, Accra P.O. Box LG43, Ghana; aablordey@noguchi.ug.edu.gh

**Keywords:** *Pseudomonas otitidis*, *Pseudomonas aeruginosa*, metallo-β-lactamases POM-1, carbapenems, virulence factors, maximum-likelihood phylogeny

## Abstract

**Introduction:** *Pseudomonas otitidis*, known for carrying the *bla*_POM-1_ gene and linked to various diseases, is widely distributed. However, its prevalence in Ghana is unknown, mainly due to misidentification or inadequate research. In this study, for the first time, we characterized *P. otitidis* from Densu river water in Ghana. **Methods:** The antimicrobial susceptibility and whole genome characteristics of two strains (Tg_9B and BC12) were determined. The resistance and virulence features were determined using ResFinder and the VFDB database, respectively. Maximum-likelihood phylogeny was conducted based on amino acid sequences of *bla*_POM-1_ and *P. otitidis* core genomes. **Results:** The strains carried *bla*_POM-1_ on the chromosome, with only Tg_9B showing intermediate resistance to meropenem. Tg_9B had a unique genetic make-up downstream of *bla*_POM-1_, compared with BC12 and other reference strains. Both strains harbored virulence factors able to induce pathogenicity through immune evasion. The efflux pump genes (*adeF*, *rsmA*, and *qacG*) were present in the genomes of all the strains used in this study. The amino acid sequences of *POM-1* in the strains shared a sequence homology with seven other sequences from different countries. **Conclusions:** This study highlights the emergence of *bla*_POM-1_ harboring *P. otitidis* in Ghana and affirms the conservation of *bla*_POM-1_ and *adeF*, *rsmA*, and *qacG* in the species.

## 1. Introduction

Metallo-β-lactamases (MBL) are well known for their clinical significance in resistance determination, attributed to their potent carbapenemase activities [1]. These enzymes are classified as class B β-lactamases, which are further subdivided into three primary structural subclasses: B1, B2, and B3 [2]. Bacterial strains of clinical significance relevant to resistance determination, such as *Bacillus* spp., *Stenotrophomonas maltophilia*, *Aeromonas* spp., *Bacteroides fragilis*, *Flavobacteria* spp., and *Pseudomonas otitidis*, are infrequently intrinsically resistant to carbapenems [3,4,5].

*P. otitidis* is a species of *Pseudomonas* and was initially documented to carry an intrinsic MBL known as POM (*P. otitidis* MBL) in a patient diagnosed with acute otitis externa [6]. The subclass B3 MBL POM-1 enzyme demonstrates broad substrate specificity, particularly towards penicillin and carbapenems [7]. MBL-producing *Pseudomonas* clones have been associated with the onset of various critical illnesses, such as septicemia and pneumonia [8]. The *bla*_POM-1_ gene harbored by *P. otitidis* encodes a carbapenem-hydrolyzing MBL [9] and is also reported to be associated with patients diagnosed with necrotizing fasciitis and peritonitis [10].

Determining the appropriate oral antibiotic for pneumonia and other infections caused by *P. otitidis* at discharge can be challenging. This was evident in a patient whose blood isolate was sensitive to piperacillin–tazobactam and gentamicin; after a 7-day treatment, they were discharged but returned a month later with bilateral pneumonia and bronchiectasis. Additionally, a *P. otitidis* isolate from a Japanese patient with multiple myeloma was initially treated with sulfamethoxazole 400 mg/trimethoprim 80 mg, but symptoms reappeared on the fourth day. The treatment was then switched to meropenem and later substituted with a combination of cefepime and levofloxacin. In Denmark, fluoroquinolones are considered the only effective oral antibiotics against *Pseudomonas* species, and it is recommended to use a combination of at least two antibiotics. Prolonged parenteral antibiotic treatment is often necessary to effectively eradicate *P. otitidis*. Nevertheless, reports concerning *P. otitidis* in Ghana were largely nonexistent until recently, when Logtong et al. documented the isolation of this species from pharmaceutical effluent [11]. However, their study did not address the genomic characteristics of the isolated strain.

The limited reporting of *P. otitidis* may be attributed to its misidentification as the important human pathogen *Pseudomonas aeruginosa*, a species recognized for its colonization capabilities, immune evasion strategies, and potential to induce lesions in human hosts [12]. This misidentification is likely to have been due to the similarities in their genotypic and phenotypic profiles, with *P. otitidis* sharing 84.3% of its virulence genes with *P. aeruginosa*. Also, PCR and MALDI-TOF/MS have been reported as reliable alternatives for bacterial identification [10]. However, many hospitals in Ghana resort to phenotypic tests due to resource limitations. This issue was confirmed in recent studies, where *P. otitidis* was misidentified as *Pseudomonas fluorescens* by the API 20E system [11]. The misidentification of pathogens can lead to significant issues, including the selection of inappropriate antibacterial therapies. This is especially critical for immunocompromised patients, who are often treated with carbapenems that are ineffective against *bla*_POM-1_-harboring *P. otitidis*. Furthermore, carbapenem resistance serves as a key indicator of a bacterium’s capacity to develop further resistance. Therefore, it is imperative to monitor and understand the presence and mechanisms of carbapenem-resistant *P. otitidis*, as this knowledge can inform infection control practices and contribute to broader efforts aimed at combating antibiotic resistance.

*P. otitidis*’s presence extends beyond clinical settings, inhabiting lagoons [13], food [14], dead bodies of animals [12], activated sludge [15,16], freshwater [13,17,18], and drinking water [19]. Rivers are recognized as fundamental sources of renewable water for humans and the freshwater biosphere [20]. However, the microbial heterogeneity in flowing freshwater is often underemphasized compared with marine or lake ecosystems [21]. The structure of a river’s microbial community has been proposed as an indicator of pollution [22] and may encompass not only broad functional diversities but also multi-drug-resistant bacteria, which could pose a pathogenic risk to humans and livestock [23].

The potential increase in pathogenic bacteria in river water is a significant concern in developing countries characterized by inadequate sewage treatment, low-income levels, rapid population growth, and water stress [24]. Therefore, accumulating the antimicrobial resistance data of water-borne pathogens in aquatic environments is required in order to monitor them, particularly in resource-limited regions. This study marks the first comprehensive genomic analysis of carbapenem-resistant *bla*_POM-1_ *P. otitidis* isolate collected from a river in Ghana.

## 2. Results

### 2.1. Antimicrobial Susceptibility Profiles

**Figure 1:**
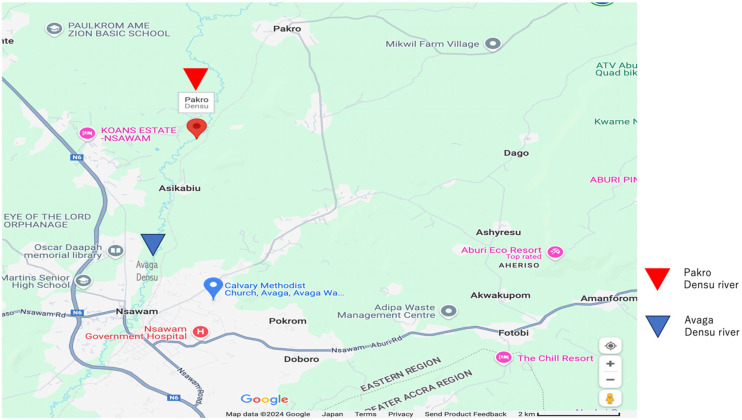
Graphical map of the sampling sites with a scale of 2 km. The red triangle denotes the location of Pakro Densu River, while the blue triangle indicates the position of Avaga Densu River.

Tg_9B and BC12 strains isolated from different parts (Avaga and Pakro) of the Densu river (Figure 1) were identified as *P. otitidis* by 16S rRNA sequencing. The Tg_9B strain showed intermediate resistance to meropenem but was susceptible to all the other antimicrobials used (Table 1). However, the BC12 strain was susceptible to all the classes of antimicrobials used (Table 1). Both strains were mCIM-negative. The results were interpreted according to the CLSI 2022 guidelines [25].

### 2.2. Genomic Characterization, Resistome, and Virulome Analysis of P. otitidis Tg_9B and BC12 Strains

The strains contained the chromosomal *bla*_POM-1_ gene, which was situated on a 6,050,884 bp chromosome (accession no. CP132337) in strain Tg_9B and on a 6,113,211 bp chromosome (accession no. CP102328.1) in strain BC12. Both strains also harbored the resistance–nodulation–cell division (RND) antibiotic efflux pump genes (*adeF* and *rsmA*) and the small multi-drug resistance (SMR) efflux pump gene (*qacG)*.

Virulence factors of Tg_9B and BC12 were investigated; one hundred and sixty (160) and one hundred and sixty-five (165) virulence factors were identified via the Virulence Factor Database, respectively (Table 2). Genes encoding quorum sensing, anti-phagocytosis, protease enzyme regulation, iron uptake, efflux pump activity, endotoxin and toxin adherence, amino acid and purine metabolism, immune evasion and invasion, magnesium uptake, stress adaption, and secretion systems were detected (Table 2 and Supplementary Table S1).

### 2.3. Phylogenetic Relationship of POM Amino Acid Sequences and P. otitidis Core Genomes

**Figure 2:**
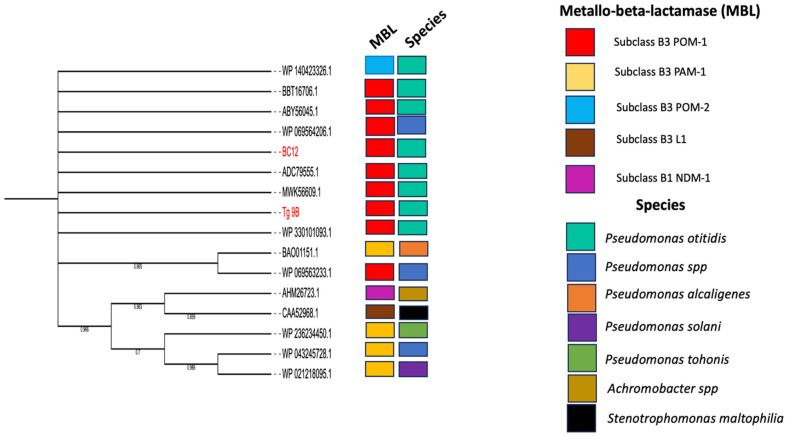
Maximum-likelihood phylogenetic tree of amino acid sequences (red highlights) isolated in this study (river water = 2), and 16 reference MBL amino acids sequences (10 POM subclass B3 MBL, 4 PAM-1 subclass B3 MBL, L1 subclass B3 MBL, and NDM-1 subclass B1 MBL) deposited to the NCBI from different sources and countries.

The maximum-likelihood phylogenetic tree was constructed using POM-1 amino acid sequences from *P. otitidis* strains Tg_9B and BC12, in conjunction with 14 subclasses of MBL amino acid sequences (Figure 2). Notably, Tg_9B and BC12 shared a sequence homology with seven POM-1 amino acid sequences (BBT16706.1, ABY56045.1, WP069564206.1, WP140423326.1, ADC79555.1, MWK56609.1, WP330101093.1) and a POM-2 enzyme (WP140423326.1) (Figure 2). Also, another POM-1 amino acid sequence (WP069563233.1) from *Pseudomonas spp.* shared a close sequence homology with a PAM-1 enzyme B3 (BAO0115.1) from *P. alcaligenes* (Figure 2). This observation corroborates prior studies indicating that PAM-1 shares a 72.4% amino acid identity with POM-1 [26]. The POM-1 amino acid sequences from our study strains were not closely related to the other subclass B3 (L1, PAM-1, and NDM-1) (Figure 2).

**Figure 3:**
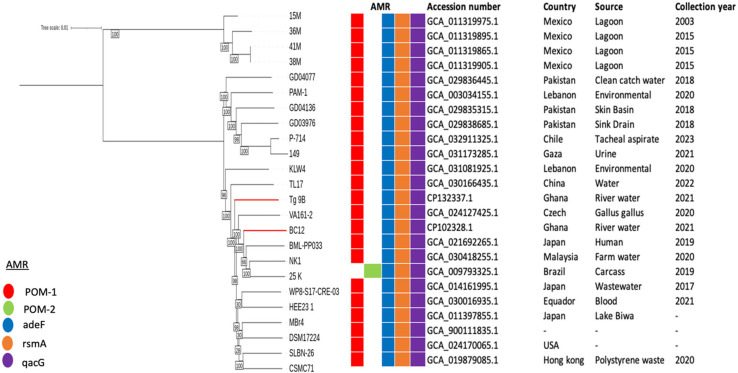
Maximum-likelihood phylogenetic tree based on *P. otitidis* core genomes. The phylogeny of Tg_9B and BC12 with respect to the other *P. otitidis* strains is highlighted with red-colored branches. The occurrence of AMR genes in these genomes is also illustrated, as well as the genome data (accession number, country, source, and year of collection).

A maximum-likelihood phylogenetic tree was constructed using the core genomes of two *P. otitidis* strains from this study and twenty-two *P. otitidis* strains obtained from the NCBI database. It was revealed that Tg_9B and BC12 shared a common lineage with TL17, VA161-2, BML-PP033, NK1, and 25 K strains, originating from China, Czech Republic, Japan, Malaysia, and Brazil, respectively (Figure 3). Interestingly, BC12 showed a close relationship with BML-PP033, isolated in Japan in 2019, while Tg_9B was closely associated with VA161-2 found in the Czech Republic in 2020 (Figure 3). Furthermore, all twenty-two strains included the presence of the RND efflux pump genes (*adeF* and *rsmA*) and SMD efflux pump gene, (*qacG)*, which were also identified in Tg_9B and BC12 (Figure 3).

### 2.4. Genetic Environment of Tg_9B and BC12 and Their Associated Virulence Factors

**Figure 4:**
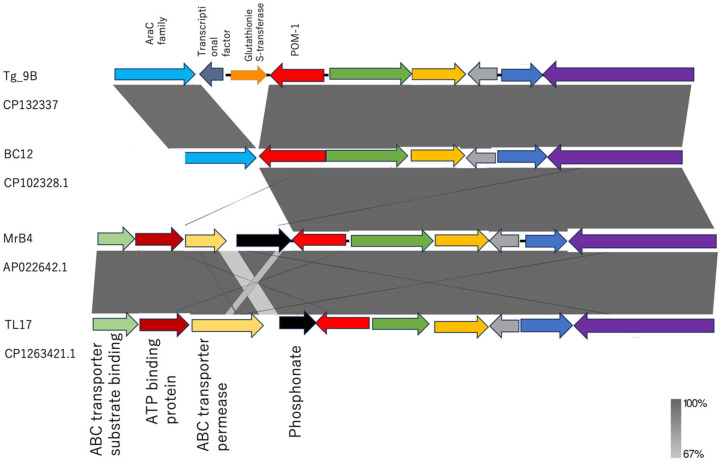
Linearized comparison of the *bla*_POM-1_ genetic environment of our study strains (Tg_9B, BC12) and strains obtained from the NCBI database (MrB4 and TL17). Similar features are represented by the same color. Gray shading indicates regions of shared homology among different elements. The darker and lighter shadings represent 100% and 67% homology, respectively.

Upstream of *bla*_POM-1_, both strains exhibited the same cascade of open reading frames (Figure 4). Interestingly, downstream of *bla*_POM-1_, strain Tg_9B showcased gene sequences of *glutathione S-transferase (GST) gene*, *transcriptional factor (TF) gene*, and *araC*, whereas strain BC12 only contained *araC* (Figure 4).

In a detailed analysis of the genetic environments of Tg_9B and BC12 in comparison to the *bla*_POM-1_ chromosomal genomes from strains MBr4 (accession no. APO2264.1) and TL17 (accession no. CP1263421.1) retrieved from the NCBI database, a striking contrast was revealed. While MBr4 and TL17 exhibited a shared genetic arrangement with Tg_9B and BC12 upstream of *bla*_POM-1_, the downstream sequences showcased a distinct composition of genes of *phosphonate*, *ABC transporter permease*, *ATP binding protein*, and *ABC transporter substrate binding protein*, setting them apart from Tg_9B and BC12 (Figure 4).

## 3. Discussion

Water, a natural habitat for *Pseudomonas* spp., provides a diverse environment for their adaptation and reproduction, spanning rivers, tap water, seawater, and bottled drinking water [27]. Likewise, *P. otitidis* has been identified in wastewater, highlighting the conservation of *bla*_POM-1_ within the species [4]. This study focused on river water in Ghana, a vital resource for humans and livestock, and a site of recreation, particularly for children.

The *bla*_POM-1_ gene produces an MBL that confers resistance to carbapenems. However, the gene’s resistance to carbapenem varies, mostly in the case of imipenem and meropenem. While resistant to imipenem and meropenem, it remains susceptible to piperacillin–tazobactam, ceftazidime, and aztreonam, as per CLSI or EUCAST guidelines [4]. Some strains may show sensitivity to imipenem but reduced susceptibility to meropenem, as seen in a study by Vieira et al. in 2020 [12]. This pattern was observed in one strain in the current study. While BC12 was susceptible to all antimicrobials, strain Tg_9B showed intermediate resistance to meropenem. Furthermore, a study by Wong et al. in 2015 [14] found that *P. otitidis* strains from food were generally sensitive to all the antimicrobials tested, except for imipenem and meropenem. Thaller et al. collected 20 strains of *P. otitidis* to investigate their sensitivity to metallo-β-lactams and the production of metallo-β-lactamases (MBLs). That study found that all strains exhibited sensitivity to piperacillin, cefotaxime, ceftazidime, and aztreonam, while some strains demonstrated a decrease in sensitivity to carbapenems. POM-1 has also been reported to have a higher catalytic efficiency against carbapenems compared with piperacillin, ceftazidime, or aztreonam [7]. This implies that immunocompromised patients who are typically treated with carbapenems require comprehensive investigation to ascertain that their infections are not attributable to *bla*-_POM-1_-producing *P. otitidis*. Both strains harbor the *bla*_POM-1_ gene, known for its lack of hydrolyzing activity towards aztreonam, like other MBLs [28], and this was confirmed as they displayed susceptibility to aztreonam treatment. This underscores the clinical potential of aztreonam in the management of infections caused by MBL-producing bacterial pathogens [29].

The *bla*_POM-1_ gene is conserved in *P. otitidis*, a species that is widely distributed and has been implicated in numerous infections. For instance, Kim et al. (2016) documented two critical cases: one involving necrotizing fasciitis and the other peritonitis [10]. In 2015, researchers isolated 10 strains identified as *P. otitidis* from foot cleft patients [30]. In 2020, Japanese researchers sequenced the whole genome of *P. otitidis* TUM18999 from burn patients [31]. In 2021, Danish physicians reported a case involving a patient with moderate chronic obstructive pulmonary disease, bronchiectasis, and recurrent pneumonia, where blood cultures demonstrated growth of *P. otitidis*. The study reported that this emerging pathogen poses a risk of misdiagnosis, which is concerning [32]. Furthermore, *P. otitidis* is prevalent in natural environments. Miyazaki et al. isolated a *P. otitidis* strain from Lake Biwa, Japan, and sequenced its genome [18]. Kaur et al. studied tap water from public toilets in Punjab, India, isolating 25 bacterial strains, including *P. otitidis* [33]. Additionally, researchers identified 13 isolates as *P. otitidis* from carbapenemase-producing Gram-negative bacilli in American factories and adjacent wastewater [34]. Vieira et al. isolated meropenem-insensitive *P. otitidis* from chicken carcasses and conducted in-depth genomic characterization [12]. Alarmingly, carbapenem-resistant *P. otitidis* has also been isolated from frozen food. Given these findings, it is not surprising that our maximum-likelihood phylogenetic analysis revealed that the genes from Ghana identified in our study shared sequence homology with *bla*_POM-1_ genes identified in seven other *P. otitidis* strains from various countries (Figure 2). This confirms the high conservation and species-specific nature of *bla*_POM-1_, while also indicating significant sequence variability for the POM-1 amino acid among *P. otitidis* strains. However, the prevalence of the *bla*_POM-1_ gene in Ghana is currently unknown, making this study crucial for raising awareness among clinicians and microbiologists and highlighting the necessity for further research.

Virulence factors in *P. aeruginosa* disrupt host cell signaling pathways and target the extracellular matrix [35], contributing to various diseases by evading the host’s immune system [36]. The bacterial LPS, consisting of lipid A and O antigens [37], triggers immune responses leading to dysregulated inflammation, potentially causing morbidity and mortality [38]. *P. aeruginosa’s* elastase LasB destroys host tissue, crucial in burn wounds and acute lung infections [39], while alginate reduces susceptibility to phagocytosis [40]. Notably, all these virulence factors were found in both *P. otitidis* strains. This reflects previous reports that *P. otitidis* shared 83.4% of the virulence factors produced by *P. aeruginosa* [12]. Kim et al. highlighted *P. otitidis* as a potential cause of hospital-acquired infections [10]. The isolation of these strains from river water, a source of drinking water for humans and livestock, raises significant concerns and emphasizes the need for preventive measures. This result indicates that *bla*_POM-1_-producing *P. otitidis* isolates, either carbapenem-resistant or -susceptible, are believed to possess the ability to actively participate in immune evasion mechanisms, potentially contributing to severe outcomes such as mortality.

The global prevalence of *bla*_POM-1_-producing *P. otitidis* is well documented, but Ghana has yet to contribute to this research area. There was a report of two *P. otitidis* isolates harboring the *bla*_POM-1_ gene in Nigeria [41]; however, the significance of this discovery was not extensively highlighted. To bridge this gap, we collected twenty-two *P. otitidis* strains from the NCBI database and integrated our two river strains from Ghana into a comprehensive maximum-likelihood phylogenetic analysis. The discovery that Tg_9B and BC12 share a common genetic ancestry with strains from China, Czechia, Japan, Malaysia, and Brazil (Figure 3) underscore the extensive distribution of *P. otitidis*. Given its ubiquitous nature, it is expected that more species may be present in Ghana but perhaps misidentified due to their shared genotypic and phenotypic characteristics with *P. aeruginosa*. The challenges in accurately identifying *P. otitidis* using phenotypic tests are well documented, with PCR and MALDI-TOF/MS emerging as more reliable alternatives [10]. However, it is noteworthy that many hospitals in Africa, particularly in Ghana, continue to utilize primarily phenotypic tests for identification purposes. Therefore, it is essential to consider *P. otitidis* when a suspected *Pseudomonas* species shows unique susceptibility, such as sensitivity to penicillin or cephalosporin antibiotics, but resistance to carbapenems.

The RND efflux pump superfamily, predominantly linked to intrinsic resistance in Gram-negative bacteria, is instrumental in the emergence of multi-drug resistance. *P. aeruginosa* is characterized by 12 distinct RND pumps that share antibiotic substrates [42]. In the context of this study, both *P. otitidis* strains were found to harbor the RND pump genes *adeF* and *rsmA*, as well as the SMR efflux protein gene *qacG*, recognized for its multidrug resistance properties. The study suggests that, along with *bla*_POM-1_, the RND efflux pump genes (*adeF* and *rsmA*) and the SMD efflux pump gene (*qacG*) are essential conserved elements in *P. otitidis*. The presence of these genes in both strains from this study and in all twenty-two *P. otitidis* strains analyzed in the phylogenetic study highlights their significance and conservation within the species.

Following the divergence of *P. otitidis* from other species, the gene *bla*_POM-1_ was believed to have been acquired through horizontal transfer mechanisms and integrated downstream of the conserved phosphonate operon [12]. This genetic arrangement was observed in the reference strains (MrB4 and TL17) obtained from the NCBI database (Figure 4); however, the downstream of *bla*_POM-1_ was followed by gene sequences of *GST-TF-araC* and solely *araC* in the Tg_9B and BC12 strains, respectively (Figure 4). The protective function of *GST* against the toxic effects of antimicrobial agents lies in its ability to efficiently sequester antibiotics [43]. Due to the absence of *GST* in the BC12 strain, it can be suggested that *GST* might have coupled with *bla*_POM-1_ or played a key role in the meropenem resistance conferred by the Tg_9B strain; however, the molecular mechanisms are yet unknown. Further studies are therefore needed to unravel the association between *GST* and *bla*_POM-1_ in carbapenem resistance.

## 4. Materials and Methods

### 4.1. Sample Collection Site and Bacterial Isolation

A volume of 2000 mL of Densu river water was collected from Avaga (N 5°49′40″ W 0°20′3) and Pakro (N 5°54′42″ W 0°19′14) (Figure 1), near Nsawam, away from animal farms and medical facilities, in the eastern part of Ghana. The water samples were filtered sequentially using different sizes (5 µm, 0.8 µm, and 0.45 µm) of filters (Merck Mllipore Ltd., County Cork, Ireland). The filters were put in 1 mL of sterile saline, and a volume of 100 µL was enriched in 2 mL tryptic soy broth (TSB) (Oxoid Ltd., Basingstoke, UK) at 37 °C overnight. A volume of 20 µL of the bacterial cultures was spread on bromothymol blue agar (Eiken Chemical Co., LTD, Tochigi, Japan,) plates. About five to eight morphologically different colonies from each plate were further passaged onto fresh agar plates and stored in skimmed milk (Morinaga Milk, Tokyo, Japan) for further analysis.

### 4.2. 16 S rRNA Species Identification

Crude DNA was extracted from the isolates using a Nucleospin Tissue Kit (Macherey-Nagel, Düren, Germany) as described previously [44]. Ten (10 µL) microliters of PCR reaction mix was prepared with 5 µL of 2× Emerald premix (Takara, Japan), 0.5 µL each of forward primer 8UA (5′-AGAGTTTGATCMTGGCTCAG-3′) and reverse primer 1485B (5′-TACGGTTACCTTGTTACGAC-3′), 3 µL of nuclease-free water, and 1 µL of DNA template. The PCR was run at an initial denaturation at 98 °C for 1 min; followed by 30 cycles of 98 °C for 5 s, 57 °C for 10 s, 72 °C for 1 min, and a final extension at 72 °C for 3 min. The PCR products were purified with EXOSAP IT (Applied Biosystems, Thermo Fisher Scientific, Waltham, MA, USA) and sequenced on a 3730xl DNA Analyzer (Thermo Fisher Scientific) with a BigDye Terminator v3.1 Cycle Sequencing Kit (Thermo Fisher Scientific) with 100% coverage, to generate at least 1400 bp. Identification was performed via BLAST searches of the NCBI database, utilizing a threshold identity of 100% and an e-value cutoff of 0.05, in accordance with the results obtained from sequencing.

### 4.3. Antimicrobial Susceptibility Testing and Bla_POM-1_ Genes Screening

The minimum inhibitory concentrations (MICs) of antibiotics were determined by broth micro-dilution using DP45 dry plates (Eiken Chemical Co., Tokyo, Japan) as described in the CLSI M100-S30 guidelines [25]. Isolates were further screened for carbapenemase production following the modified carbapenemase inactivation method as outlined in the CLSI guidelines.

### 4.4. Genome Sequencing and Analysis

For short-read sequencing, genomic DNA was extracted with a Magattract HMW DNA kit (Qiagen, Hildon, Germany) according to the manufacturer’s instructions. Libraries were prepared using Illumina DNA prep with the IDT for Illumina DNA/RNA UD Indexes and sequenced on an Illumina MiniSeq software v4.0 (Illumina Inc, San Diego, CA, USA), generating reads of length 150 bp. Short reads were assessed for quality using fastqc v0.11.9 (https://github.com/s-andrews/FastQC (accessed on 10 September 2022)). Reads were trimmed and filtered with fastp v0.23.1 (https://github.com/OpenGene/fastp (accessed on 10 September 2022)). Libraries for long-read sequencing were prepared using the native barcoding and ligation sequencing kits EXP-NB104 (Oxford Nanopore Technologies, Oxford, UK) and SQK-LSK109 (Oxford Nanopore Technologies, Oxford, UK), respectively, according to the manufacturer’s manuals. Libraries were loaded onto FLO-MIN106 R9.4.1 flow cells and sequencing was conducted on a MinION Mk 1B (Oxford Nanopore Technologies, Oxford, UK). The generated barcoded raw reads were basecalled using Guppy v1.1.4 (https://community.nanoporetech.com/protocols/Guppy-protocol/ (accessed on 15 November 2022)). Demultiplexing of reads and trimming of adapter sequences was performed using Porechop v0.2.4 [26]. Short reads with low quality (MinION Q < 10; and long reads (≤1000 bp) were filtered out with Filtlong v0.2.1 (https://github.com/rrwick/Filtlong (accessed on 15 November 2022)). Hybrid assembly of long-read and short-read sequences was carried out with Unicycler v0.4.8 [45]. Genomes were uploaded into the RAST server (https://rast.nmpdr.org/ (accessed on 22 November 2022)) for annotation. Assembled genomes were screened for acquired antimicrobial-resistance genes, sequence types (STs), and plasmid replicon types, through the web-based CGE databases ResFinder v4.6.0 (https://cge.food.dtu.dk/services/ResFinder/ (accessed on 3 may 2024)), MLST v2.0.9 (https://cge.food.dtu.dk/services/MLST/ (accessed on 15 January 2023)), and Plasmid Finder v2.0.1 (https://cge.food.dtu.dk/services/PlasmidFinder/ (accessed on 15 January 2023)), respectively. Antimicrobial-resistance genes were also identified through the web-based comprehensive antibiotic resistance database (CARD) using the resistance gene identifier RGI 6.0.3, CARD 3.3.0 (https://card.mcmaster.ca/analyze/rgi (accessed on 3 May 2024)). Virulence factors were identified using the VirulenceFinder v2.0.5 (http://www.mgc.ac.cn/cgi-bin/VFs/v5/main.cgi (accessed on 15 March 2024)). Complete genomes of the *P. otitidis* strains identified in this study have been deposited in NCBI under BioProject PRJNA473419. Genetic context and comparison of the identified *bla*_POM-1_ gene structures were carried out using Easy Fig v.2.2.2.

### 4.5. Phylogenetic Analysis

The *bla*_POM-1_ amino acid sequences of Tg_9B and BC12 were compared with 16 partial and full amino acid sequences that were downloaded from the NCBI database. Evolutionary analyses were conducted in MEGA v11.0 [46]. The evolutionary history was inferred by using the maximum-likelihood method and JTT matrix-based model [47] with a bootstrap of 1000 replicates [48]. To investigate the strains within a global context, a maximum-likelihood phylogenetic tree was constructed, incorporating 2 strains and 22 *P. otitidis* genomes obtained from the NCBI database. Pangenome analysis was conducted utilizing Roary v3.12.0 [49]. The resultant core-genome alignment file from Roary was utilized in Iqtree to generate a phylogenetic tree with 1000 bootstrapping replicates [50]. The visualization and annotation of the trees were executed using iTOLv4.0, as outlined previously [51]. The lists of reference MBL amino sequences and *P. otitidis* genomes are summarized in Supplementary Tables S2 and S3, respectively.

## 5. Conclusions

Initially acknowledged for its involvement in otic infections, *P. otitidis*, known for its ubiquitous nature, was identified in river water in Ghana in the present study. This marks the first documented case of carbapenem-resistant *bla*_POM-1_-producing *P. otitidis* in Ghana. The challenges in accurately identifying *P. otitidis* using phenotypic tests, particularly in low- and middle-income countries, raise significant concerns.

The detection of *bla*_POM-1_ in the pathogen from river water underscores the potential environmental dissemination of this MBL subclass. It is essential for clinicians and microbiologists to be cognizant of the potential of *P. otitidis* to cause severe infections, especially in immunocompromised hospitalized patients. Further research involving a larger number of isolates is warranted to ascertain the true prevalence of the species in Ghana and to elucidate the unknown molecular mechanisms contributing to *bla*_POM-1_-mediated carbapenem resistance.

## Figures and Tables

**Table 1:** Minimum Inhibitory Concentration (MIC) profile of *P. otitidis* Tg_9B and BC12.

Antimicrobial Agents	Breakpoint for Resistance (µg/mL)	Tg_9B (µg/mL)	BC12 (µg/mL)
Piperacillin	≥128	4	4
Piperacilli–tazobactem	≥128/4	4/4	4/4
Cefipime	≥32	1	≤0.5
Ceftazidime	≥32	2	2
Gentamicin	≥16	≤1	≤1
Amikacin	≥64	≤4	≤4
Levofloxacin	≥4	2	≤0.5
Aztreonam	≥32	4	8
Imipenem	≥8	2	2
Meropenem	≥8	4	≤0.5
Ciprofloxacin	≥2	≤0.5	≤0.25
Tobramycin	≥16	≤1	≤1
Colistin	≥4	≤1	<1

**Table 2:** Epidemiological Genomic data of Tg_9B and BC12.

Virulence Factors	Number of Related Genes	
	Tg_9B	BC12
Adherence	77	79
Anti-phagocytosis	23	24
Enzyme	2	2
Iron uptake	16	16
Protease	1	1
Quorum sensing	1	1
Regulation	4	3
Secretion system	23	24
Toxin	1	2
Amino acid and purine metabolism	1	1
Efflux pump	1	1
Endotoxin	1	1
Glycosylation system	1	1
Immune evasion	2	3
Invasion	2	2
Magnesium uptake	1	1
Serum resistance	1	1
Stress adaption	2	2

## Data Availability

The datasets presented in this study can be found in online repositories at the National Center for Biotechnology Information (NCBI) BioProject database under accession number PRJNA473419. The accession numbers for the BioSamples of Tg_9B and BC12 are SAMN36900137 and SAMN30101387, respectively.

## Supplementary Materials

The following supporting information can be downloaded at: https://www.mdpi.com/article/10.3390/antibiotics14010050/s1. Table S1: Virulence profiles of *P. otitidis* in this study, Table S2: List of MBL amino-acid sequences downloaded from NCBI GenBank database, Table S3: List of *P. otitidis* genomes downloaded from GenBank Database, Table S4: Annotation statistics of BC12 and Tg_9B, Table S5: Antimicrobial resistant determinants of Tg_9B using Resfinder, Table S6: Antimicrobial resistant determinants of Tg_9B using CARD, Table S7: Antimicrobial resistant determinants of BC12 using Resfinder, Table S8: Antimicrobial resistant determinants of BC12 using CARD.
